# Chemically Modified Bovine β-Lactoglobulin as a Broad-Spectrum Influenza Virus Entry Inhibitor with the Potential to Combat Influenza Outbreaks

**DOI:** 10.3390/v14092055

**Published:** 2022-09-16

**Authors:** Yuhong Fu, Peiyu Li, Wei Xu, Zezhong Liu, Cong Wang, Qian Wang, Jiayi Tang, Weihua Li, Lu Lu, Shibo Jiang

**Affiliations:** 1 Key Laboratory of Medical Molecular Virology of MOE/MOH, School of Basic Medical Sciences, Shanghai Public Health Clinical Center, Fudan University, 131 Dong An Rd., Xuhui District, Shanghai 200032, China; 2 Department of Infectious Diseases and Shenzhen Key Laboratory for Endogenous Infection, Shenzhen Nanshan People’s Hospital and the Affiliated Shenzhen Sixth Hospital of Guangdong Medical University, Shenzhen 518052, China; 3 NHC Key Lab of Reproduction Regulation (Shanghai Institute for Biomedical and Pharmaceutical Technologies), Fudan University, 2140 Xie Tu Rd., Xuhui District, Shanghai 200032, China

**Keywords:** influenza virus, viral entry inhibitor, broad-spectrum, prevention, chemical modification, bovine β-lactoglobulin

## Abstract

Frequent outbreaks of the highly pathogenic influenza A virus (AIV) infection, together with the lack of broad-spectrum influenza vaccines, call for the development of broad-spectrum prophylactic agents. Previously, 3-hydroxyphthalic anhydride-modified bovine β-lactoglobulin (3HP-β-LG) was proven to be effective against human immunodeficiency virus (HIV) and severe acute respiratory syndrome coronavirus 2 (SARS-CoV-2) and it has also been used in the clinical control of cervical human papillomavirus (HPV) infections. Here, we show its efficacy in potently inhibiting infection by divergent influenza A and B viruses. Mechanistic studies suggest that 3HP-β-LG binds, possibly through its negatively charged residues, to the receptor-binding domain in the hemagglutinin 1 (HA1) subunit in the HA of the influenza virus, thus inhibiting the attachment of the HA to sialic acid on host cells. The intranasal administration of 3HP-β-LG led to the protection of mice against challenges by influenza A(H1N1)/PR8, A(H3N2), and A(H7N9) viruses. Furthermore, 3HP-β-LG is highly stable when stored at 50 °C for 30 days and it shows excellent safety in vitro and in vivo. Collectively, our findings suggest that 3HP-β-LG could be successfully repurposed as an intranasal prophylactic agent to prevent influenza virus infections during influenza outbreaks.

## 1. Introduction

Influenza A virus (IAV) can cause pandemics of influenza, such as those that were caused by H1N1 in 1918, H2N2 in 1957, H3N2 in 1968, and H1N1 in 2009, resulting in millions of deaths worldwide (https://www.cdc.gov/flu/pandemic-resources/basics/past-pandemics.html, accessed on 30 July 2022). Vaccination is recommended as a main preventive strategy to combat influenza epidemics or pandemics [1]. However, the effectiveness of influenza virus vaccines is limited by drift variants and the currently available influenza vaccines are strain-specific. This means that vaccines that are effective against seasonal influenza viruses are generally ineffective against highly pathogenic strains, such as the influenza A(H7N9) and A(H5N1) viruses [2]. Research that is devoted to the development of universal influenza vaccines against divergent IAVs has taken decades, but it remains unclear whether such a universal vaccine is achievable. The sudden outbreaks that are caused by the rapid spread of emerging IAVs make it almost impossible to develop and produce a timely and effective vaccine to combat the resulting epidemic or pandemic [3]. Therefore, it is essential to develop new strategies, such as antiviral agents, to stop the spread of the virus and control a potential outbreak.

The envelope protein hemagglutinin, of IAVs, consists of two disulfide-linked subunits, HA1 and HA2, which are responsible for the binding of the virion to a sialic acid receptor on the host cell and membrane fusion in the endosome with low pH, respectively [4,5,6]. Therefore, HA is an important target for the development of virus entry inhibitor-based anti-influenza drugs [7,8,9,10].

The currently available oral anti-influenza virus drugs, including M2 ion-channel inhibitors (e.g., amantadine and rimantadine) and neuraminidase inhibitors (such as oseltamivir and zanamivir) may not be suitable for the prevention of infection by IAVs with multiple drug resistance [11,12,13]. Therefore, it is necessary to develop an intranasally applicable antiviral with a broad-spectrum antiviral effect by targeting viral HA in order to prevent IAV infection at the first sign of an outbreak.

Our previous studies have shown that 3-hydroxyphthalic anhydride (3HP)-modified β-lactoglobulin (β-LG), 3HP-β-LG, could inhibit the infection of human immunodeficiency virus (HIV), human papillomavirus (HPV), and severe acute respiratory syndrome coronavirus 2 (SARS-CoV-2). Entry of the virus into host cells is blocked as a result of the binding between the negatively charged residues of 3HP-β-LG and the positively charged residues on the surface proteins of virions [14,15,16,17,18,19]. We thus hypothesized that 3HP-β-LG might use a similar mechanism to block the entry of IAVs, thus inhibiting IAV infection.

In this study, we demonstrated that 3HP-β-LG could effectively inhibit the infection by divergent influenza viruses of MDCK cells by its interaction with the virions, rather than the host cells. Moreover, 3HP-β-LG was effective in protecting *C57BL/6 mice* from challenges with lethal dose of influenza A(H1N1), A(H3N2), and A(H7N9) viruses. Its safety profile, both in vitro and in vivo, has been verified. Therefore, 3HP-β-LG is a candidate for further development as an intranasally administered prophylactic that may be used to prevent infection by divergent emerging influenza viruses.

## 2. Materials and Methods

### 2.1. Cells, Viruses, Plasmids, and Mice

MDCK, HEK293T, Vero, A549, Huh-7, and U87 cell lines were purchased from the American Type Culture Collection (ATCC). The cells were grown in Dulbecco’s Modified Eagle Medium (DMEM), containing 10% fetal bovine serum (FBS) and 100 IU/mL penicillin and streptomycin each, and they were cultured in an incubator at 37 °C with 5% CO_2_.

Influenza A viruses, including A/Shanghai/4664T/2013(H7N9), A/Guizhou/54/89(H3N2), A/Puerto Rico/8/34(H1N1), and A/Shanghai/37T/2009(H1N1), and influenza B viruses, including B/Florida/2006 and B/Brisbane/2008, were obtained from BEI Resources [20,21]. A/Shanghai/4664T/2013(H7N9), A/Guizhou/54/89(H3N2), and A/Puerto Rico/8/34(H1N1) were grown in chicken embryos, while the others were grown in MDCK cells.

Plasmids *pVKD-HA* (GenBank accession no. KC853228) and *pVKD-NA* (GenBank accession no. KC853231) that were isolated from the influenza A/Shanghai/4664T/2013 (H7N9) virus were obtained from the Shanghai Public Health Clinical Center [22,23]. Plasmid *pNL4-3.Luc.R^−^E^−^* was bought from BioVector NTCC. Plasmids *H5N1 (A/QH/59/05) HA* (GenBank accession no. ABE68921), *H5N1 (A/Hong Kong/156/97) HA* (GenBank accession no. AF036356.1), *H5N1 (A/Thailand/Kan353/2004) HA* (GenBank accession no. EF541411.1), and *H5N1 (A/Thailand/Kan353/2004) N1-typed NA* were constructed as has been previously described [20,21].

*C57BL/6 mice* were purchased from Shanghai SLAC Experimental Animal Co., Ltd. The mice that were used for the H7N9 challenge study were maintained in the Animal Biosafety Level 3 Laboratory (ABSL-3) of Fudan University, while those that were used for the H1N1 and H3N2 challenge studies were maintained in the Biosafety Level 2 Laboratory (BSL-2). All of the animal experimental protocols were approved by the Committee on the Ethics of Animal Experiments of the School of Basic Sciences, Fudan University (Permission code: 20180302-019; permission date: 2 March 2018).

### 2.2. Preparation of 3HP-β-LG and Determination of Modification Rates of Lys and Arg with TNBS Assay and ρ-HPG Assay

The lysine (Lys) and arginine (Arg) in the β-LG (Sigma-Aldrich, Cat. No. L3908) were detected by using a 2,4,6-trinitro-benzene sulfonic acid (TNBS, Sigma-Aldrich, St. Louis, MO, USA) assay and a ρ-hydroxyphenylglyoxal (ρ-HPG, Thermo Fisher Scientific, Valley Park, CA, USA) assay, respectively, as have been previously described [24,25]. For quantitating the Lys in the 3HP-β-LG or β-LG, 25 μL protein (90 μM) was mixed with 25 μL Na_2_B_4_O_7_ for 5 min at room temperature and then with 10 μL TNBS for 5 min. The termination buffer (0.1 M NaH_2_PO_4_ and 1.5 mM Na_2_SO_3_) was added so as to stop the reaction. The absorbance at 420 nm (A420) was measured with the Tecan Infinite F200/M200 Multifunctional Microplate Reader. For quantitating the Arg residues in the 3HP-β-LG or β-LG, 90 μL protein (90 μM) was mixed with 10 μL ρ-HPG (50 mM) at room temperature for 1.5 h (avoiding light). The absorbance at 340 nm (A340) was measured as is described above. The Arg and Lys modification rates were calculated and the rate of the positively charged amino acids that were modified against the concentration of 3HP-β-LG for inhibiting viral infection was plotted as has been previously described [24,25,26].

### 2.3. Pseudotyped Influenza Virus Inhibition Assay

Pseudotyped influenza viruses (PsV) were constructed and their infectivity was evaluated as has been previously described [27]. Briefly, 3HP-β-LG and PsV were mixed and incubated for 0.5 h at room temperature. Then, the mixture was added to MDCK cells that had been cultured in a 96-well plate. The cells were then cultured in an incubator at 37 °C with 5% CO_2_ for 6 h. The cells were washed twice with PBS and further cultured in a fresh DMEM medium for 48 h. The luciferase activity was measured using the Luciferase Assay System (Promega Corp, Madison, WI, USA) with a microplate luminometer (Ultra 384, Tecan, Morrisville, NC, USA).

### 2.4. Plaque Reduction Assay

A plaque reduction assay, for measuring the inhibitory activity of 3HP-β-LG on the plaque formation that is induced by an influenza virus infection, was conducted as has been previously described [28]. Briefly, the MDCK cells that had been precultured overnight in 12-well culture plates were washed with PBS and incubated with an influenza virus at ~0.01 MOI in the presence or absence of 3HP-β-LG or β-LG (as a control) at the indicated concentration for 2 h at 37 °C. The cells were washed with PBS and covered with 1% Ultra Pure™ low melting point agarose (Invitrogen, Carlsbad, CA, USA) with 1 μg/mL TPCK-trypsin. After 2–3 days, the cell monolayer was fixed with 4% paraformaldehyde, containing 0.5% crystal violet, for 1 h and the plaque-forming units were counted.

### 2.5. Hemagglutinin Inhibition (HI) Assay

The HI assay was performed in order to measure the inhibitory activity of 3HP-β-LG or β-LG (as a control) on the infection, by the A(H1N1)/SH virus, of the MDCK cells. Briefly, serially diluted 3HP-β-LG was incubated with the A(H1N1)/SH virus for 1 h at room temperature and this step was then followed by the addition of the mixture to the MDCK cells. After 24 h, 50 µL culture supernatant was transferred to a well of a plate before the addition of 50 µL 1% fresh chicken red blood cells in PBS. HI was observed and recorded after 1h at room temperature as was previously described [28].

### 2.6. Enzyme-Linked Immunosorbent Assay (ELISA)

In order to determine whether intranasally applied 3HP-β-LG could elicit antibodies, we intranasally administered 20 μL of 3HP-β-LG or β-LG (as a control) at 40 mg/kg to C57BL/6 female mice aged 5–6 weeks (*n* = 3). The bodyweights of the mice were recorded daily. Their sera were collected 21 days post-administration in order to detect anti-3HP-β-LG antibodies using ELISA. Briefly, 3HP-β-LG or β-LG at a concentration of 5 μg/mL was immobilized on high binding-capacity 96-well plates (Corning Costar, Acton, MA, USA) at 4 °C overnight and then blocked with 5% dried skimmed milk at 37 °C for 1 h. Mouse serum at serial dilutions was added and the mixture was incubated at 37 °C for 1 h. After the plates were washed 5 times, HRP-conjugated rabbit anti-mouse antibody (1:5000) and TMB substrate were added sequentially. The absorbance was measured with the Tecan Infinite F200/M200 multifunctional microplate reader.

The binding of 3HP-β-LG with the HA protein of influenza A(H7N9) that was expressed in HEK293T cells was detected by ELISA as was previously described [27]. First, we measured the binding of the anti-3HP-β-LG antibody to 3HP-β-LG or β-LG. Briefly, 3HP-β-LG or β-LG (as a control) at 5 μg/mL was coated on wells of an ELISA plate, followed by the addition of anti-3HP-β-LG rabbit IgG (20 μg/mL) and incubation at 37 °C for 1 h. After washing, the plates were incubated with the HRP-conjugated goat anti-rabbit antibodies and TMB, sequentially, and the absorbance was measured as was described above. Next, we detected the binding of 3HP-β-LG to the HA of influenza A(H7N9). In brief, the HA protein was coated on wells of a 96-well plate at 5 μg/mL. Then, 3HP-β-LG or β-LG (as a control) at serial dilution was incubated at 37 °C for 1 h, followed by the sequential addition of rabbit anti-3HP-β-LG IgG (20 μg/mL), HRP-conjugated goat anti-rabbit antibody, and TMB. The absorbance at 450 nm (A450) was measured as was described above.

### 2.7. In Vitro Cytotoxicity Assay

The in vitro cytotoxicity of 3HP-β-LG or β-LG on MDCK, A549, U87, Vero, and Huh-7 cells was tested as has previously been described [28]. Briefly, 100 μL of MDCK cells (1 × 10^5^ cells/mL) were dispensed into the wells of a 96-well plate and incubated at 37 °C/5% CO_2_ for 24 h. The test compound at serial dilution in 100 μL of DMEM, without serum, was added to the cells. After culturing for 72 h, the cells’ viability was measured by using the Cell Counting Kit-8 (CCK-8), which was purchased from Dojindo Laboratory (Kumamoto, Japan), and by using a spectrophotometer (Ultra 384, Tecan, Morrisville, NC, USA). The percentage of cytotoxicity ((1 − % cell viability) × 100%) was calculated and the CC_50_ (half-maximal cytotoxic concentration) of the test compound was determined using Calcusyn software (Biosoft, Ferguson, MO, USA) [29].

### 2.8. Assay for Measuring In Vitro Stability of 3HP-β-LG

The inhibitory activity of 3HP-β-LG against influenza A(H7N9) PsV was examined after its storage at 4 °C, 25 °C, 37 °C, and 50 °C for 1, 2, 3, and 4 weeks, respectively. The percentage of inhibition of the 3HP-β-LG at 200 nM on influenza A(H7N9) PsV infection was measured using a luciferase assay as was described above (Section 2.3).

### 2.9. Assay for Detecting In Vivo Safety of 3HP-β-LG in Mice

Female *C57BL/6 mice* (6–8 weeks, n = 3) were intranasally administered with 40 μL of PBS (control) or 3HP-β-LG (80 or 40 mg/kg, respectively) bilaterally through their nostrils after the mice were anesthetized with pentobarbital. The protein solution was dripped into the nostrils from outside of the nostrils, which could be inhaled into the respiratory tract through the mouse’s breathing. Mouse blood was collected on day −1, day 1, day 3, and day 5. Alanine transaminase (ALT) and creatinine (CRE) were detected with ALT/GPT creatinine assay kits, respectively (Nan Jing Jian Cheng Technology, Nanjing, China). The bodyweights of the mice that were treated with 3HP-β-LG were measured and recorded daily. The mouse lungs, livers, and kidneys were also collected and placed in 10% neutral formaldehyde fixative at 4 °C. The formalin-fixed paraffin-embedded tissues were cut into 4 μm sections and stained with hematoxylin and eosin (H&E) for histological analysis.

### 2.10. Time-of-Addition Assay

In order to determine the stage of the viral life cycle that is affected by 3HP-β-LG, a time-of-addition assay was performed as has been previously described [28]. Briefly, 3HP-β-LG was added to the MDCK cells at −0.5, 0, 0.5, 1, 2, 4, 6, and 8 h, respectively, while influenza A(H7N9) PsV was added at 0 h. The cells were washed 12 h after their infection and fresh DMEM medium without FBS was added. Two days later, the luciferase activity was measured as is described above.

### 2.11. Washout Assay

In order to determine whether 3HP-β-LG acts on a protein within the host cell, a washout assay was conducted. In brief, 3HP-β-LG at the indicated concentration was added to the MDCK cells. After incubation with 3HP-β-LG at 37 °C for 1 h, the cells were either washed with DMEM or not washed. Influenza A(H1N1)/SH was added. Three days later, the inhibitory activity of 3HP-β-LG on the A(H1N1)/SH infection was assessed by the use of a CCK-8 kit. We repeated this experiment with influenza A(H7N9) PsV in the place of influenza A(H1N1)/SH and the inhibitory activity of 3HP-β-LG on the A(H7N9) PsV infection was measured by a luciferase assay as is described above.

### 2.12. Evaluation of 3HP-β-LG-Mediated Protection of Mice against Influenza Virus Infection

In order to detect the preventive efficacy of 3HP-β-LG on an influenza A(H7N9) infection, female *C57BL/6 mice* (7–10 weeks) were used. Briefly, the mice were intranasally administered with 20 μL 3HP-β-LG (*n* = 5) or β-LG (*n* = 5) at 40 mg/kg, or PBS control (*n* = 4), bilaterally through their nostrils after being anesthetized with pentobarbital. About 0.5 h later, the mice were intranasally challenged with 15 μL of a lethal dose of influenza A(H7N9), A(H3N2), or A(H1N1)/PR8. Each mouse’s bodyweight was recorded every day for 2 weeks. The lungs were observed 5 days post-infection and weighed.

In order to detect the therapeutic efficacy of 3HP-β-LG against the influenza virus challenge, the mice were intranasally challenged with 15 μL of a fatal dose of influenza A(H3N2) or A(H7N9). Four hours later, the mice were intranasally administered with 20 μL of 3HP-β-LG or β-LG at 40 mg/kg, or a PBS control. Each mouse’s bodyweight was recorded every day for 2 weeks.

### 2.13. Detection of Virus Titer in Mouse Lungs

Virus titer in the lungs of the mice that were treated with 3HP-β-LG and challenged with influenza A(H1N1)/PR8 was detected with a plaque formation assay as has previously been described. Briefly, female *C57BL/6 mice* that were 5–6 weeks old were intranasally administered with 3HP-β-LG at −0.5 h (*n* = 5) or 4 h (*n* = 4) before they were challenged with lethal influenza A(H1N1)/PR8. PBS-treated mice (*n* = 5) were included as control. On the 5th day post-infection, the mice in each group were euthanized with CO_2_ inhalation and their lungs were removed and weighed. The lung tissue was harvested and homogenized in ice-cold PBS buffer. The supernatant was collected after centrifugation for 20 min (2000 rpm) for the detection of viral titer using a plaque formation assay using MDCK cells as described above.

## 3. Results

### 3.1. The 3HP-β-LG Exhibited Potent and Broad Inhibitory Activity against Infection by Influenza A and B Viruses

**Figure 1:**
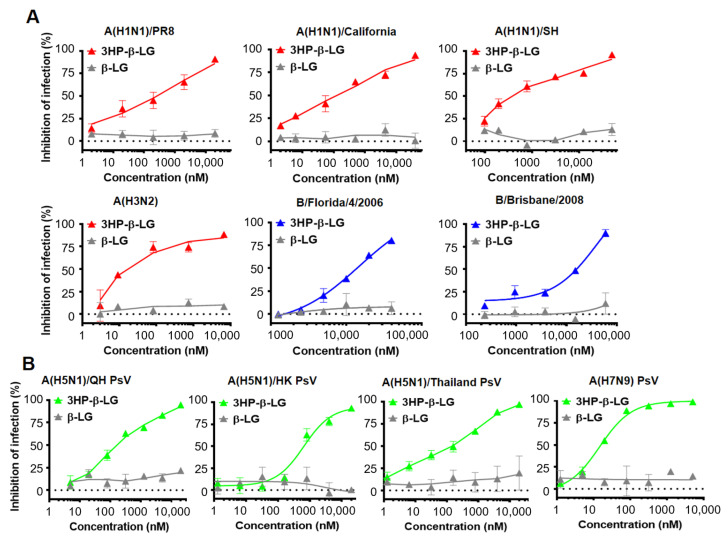
Inhibition of 3HP-β-LG against diverse influenza viruses. (**A**) Inhibition of authentic influenza viruses was detected by plaque reduction assay. (**B**) Inhibition of influenza A (H5N1) PsV infection was detected by luciferase assay. Samples were tested in triplicate and the data are presented in mean ± SD. Each experiment was repeated at least twice.

We used a plaque reduction assay to detect the inhibitory effect of 3HP-β-LG against authentic influenza virus infection in MDCK cells [28,30]. As is shown in Figure 1A, 3HP-β-LG was effective against infection by authentic influenza A(H1N1)/PR8, A(H1N1)/California, A(H1N1)/SH, A(H3N2), B/Florida/4/2006, and B/Brisbane/2008 viruses with IC50 of 3435, 165, 391, 100, 12,476, and 11,139 nM, respectively; whereas β-LG showed no detectable inhibitory activity, even at the maximum concentration of 20,000 nM (Figure 1A, Supplementary Figure S1). These results suggest that 3HP-β-LG possesses potent and broad-spectrum inhibitory activity against both influenza A and B viruses. We subsequently detected the inhibitory activity of 3HP-β-LG against infection by three influenza A(H5N1) PsVs and one influenza A(H7N9) PsV. As is shown in Figure 1B, 3HP-β-LG showed potent inhibitory activity against infection by pseudotyped influenza A(H5N1)/QH, A(H5N1)/HK, A(H5N1)/Thailand, and A(H7N9) viruses with IC50 of 117, 646, 220, and 15 nM, respectively; whereas unmodified β-LG had no detectable inhibitory activity at concentrations up to 20,000 nM, indicating that 3HP-β-LG is highly effective against infection by divergent A(H5N1) and A(H7N9) PsVs in vitro.

**Figure 2:**
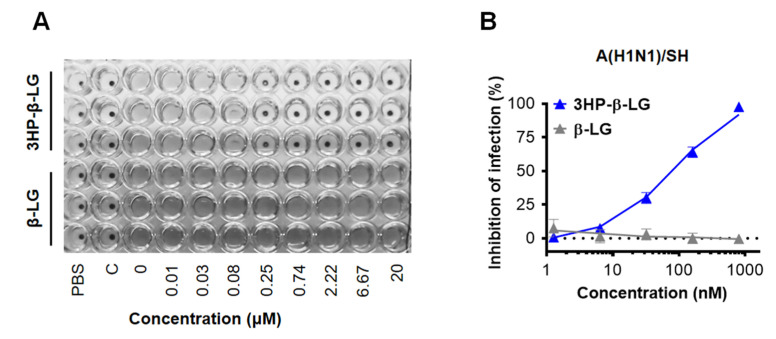
Inhibition of 3HP-β-LG against influenza A(H1N1)/SH virus infection in MDCK cells. (**A**) HI assay. (**B**) CPE reduction assay.

In order to confirm the anti-influenza virus activity of 3HP-β-LG, we also performed a hemagglutination inhibition (HI) assay and a cytopathic effect (CPE) reduction assay. We found that 3HP-β-LG was effective against the influenza A(H1N1)/SH virus infection in MDCK cells with IC50 of 133.2 ± 75.7 nM in the HI assay (Figure 2A) and 96.6 ± 4.6 nM in the CPE reduction assay (Figure 2B), respectively; whereas β-LG at the concentration of 20,000 nM had no detectable inhibitory activity.

### 3.2. The 3HP-β-LG Exhibited Good In Vitro Safety and High Stability at Different Temperatures

**Figure 3:**
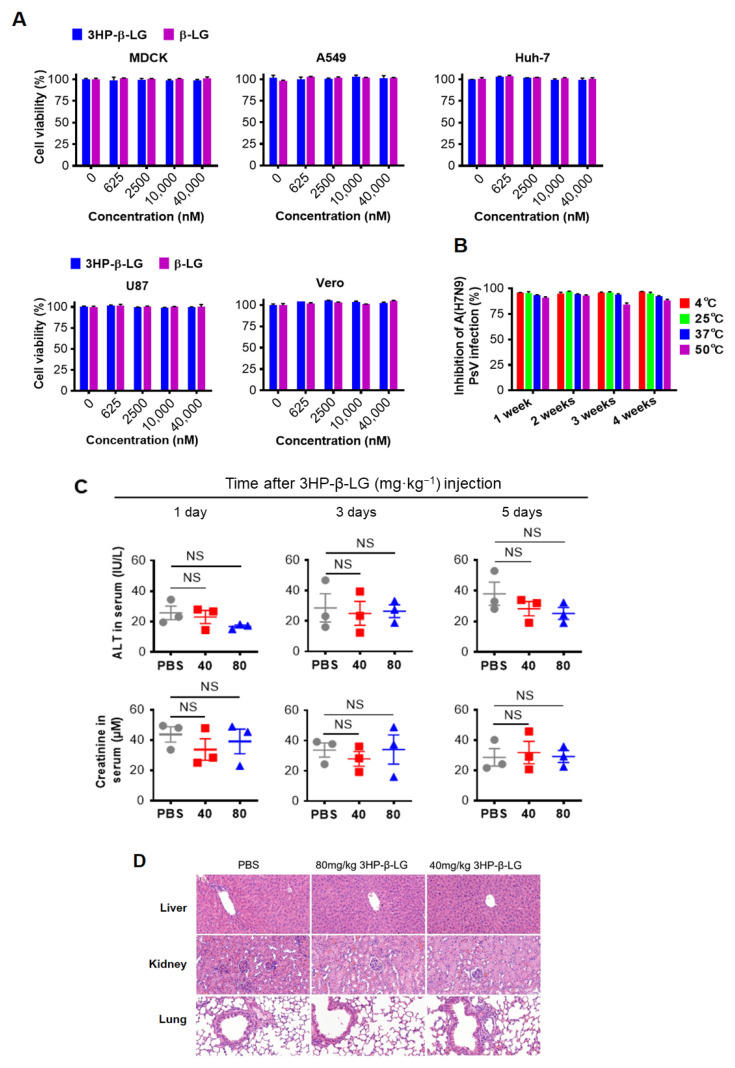
Safety in vitro and in vivo and stability of 3HP-β-LG. (**A**) Cytotoxicity of 3HP-β-LG and β-LG on MDCK, A549, Huh-7, U87, and Vero cells was detected by CCK-8 kit. (**B**) Stability of 3HP-β-LG stored at 4 °C, 25 °C, 37 °C, and 50 °C for 1, 2, 3, and 4 weeks, respectively. The percentage of inhibition of 3HP-β-LG against influenza A(H7N9) PsV was detected by luciferase assay. (**C**) The activity of ALT (IU/L) and serum creatinine in sera collected from mice at days 1, 3, and 5, respectively, after they were administered intranasally with 3HP-β-LG at 80 and 40 mg/kg, respectively, or PBS (as a control). Samples were tested in triplicate and data are presented in mean ± SD. Each experiment was repeated at least twice. NS denotes no significance. (**D**) Histopathological changes in the liver, kidney, and lungs in the mice that were administered intranasally with 3HP-β-LG at 80 and 40 mg/kg, respectively, or PBS (as control), at day 30 after the first administration of 3HP-β-LG.

In order to confirm the in vitro safety of 3HP-β-LG, we tested the cytotoxicity of 3HP-β-LG and found that neither 3HP-β-LG nor β-LG had toxicity to MDCK, A549, U87, Vero, and Huh-7 cells at concentrations as high as 40 μM (Figure 3A). In order to determine its in vitro stability, 3HP-β-LG was stored at 4 °C, 25 °C, 37 °C, and 50 °C for 1, 2, 3, and 4 weeks, respectively, before its respective inhibitory activity against influenza A(H7N9) PsV was detected. As is shown in Figure 3B, 3HP-β-LG maintained similar antiviral activity against influenza A(H7N9) PsV when it was stored at different temperatures for 1 to 4 weeks, suggesting that 3HP-β-LG is highly thermostable and can be stored and transported at regular or higher temperatures in the subtropics and even in tropical areas.

### 3.3. The 3HP-β-LG Showed High In Vivo Safety in a Mouse Model

In order to investigate in vivo safety of the present method in a mouse model, mice were administered with 3HP-β-LG and both the ALT and CRE values in their serum were measured. The results showed no significant differences in the ALT and CRE levels in the sera between the PBS control group and 3HP-β-LG treatment groups, indicating that both ALT and CRE were within the normal range (Figure 3C). The in vivo safety of 3HP-β-LG was further confirmed by the histological examination of the H&E-stained slices of mouse organs (liver, kidney, and lung). Neither infiltration of the inflammatory cells nor a chronic pathological change was found in the organs of the mice that were treated with 3HP-β-LG at 80 and 40 mg/kg, respectively (PBS as control) (Figure 3D). The bodyweights of the mice that were intranasally administered with 3HP-β-LG, β-LG at 80 mg/kg, or PBS (as a control), were monitored every day for 14 days. All of the mice showed a normal and slow growth trend and no significant difference in the bodyweights of the mice was noted among the three groups (Supplementary Figure S2A). On day 14 post-administration, the sera of the mice in these three groups were collected for the detection of antibodies against β-LG using ELISA. As is shown in Supplementary Figure S2B, a low-level of anti-β-LG antibodies was detected in the sera of the mice that were treated with 3HP-β-LG, which is not significantly different from the antibodies that were detected in the sera of the β-LG- and PBS-treated mice, indicating that the local use of 3HP-β-LG in the respiratory tract does not induce high-titer anti-β-LG antibody.

### 3.4. The 3HP-β-LG Inhibited Influenza Virus Infection by Targeting the Early Stage of Virus Entry into the Host Cell

**Figure 4:**
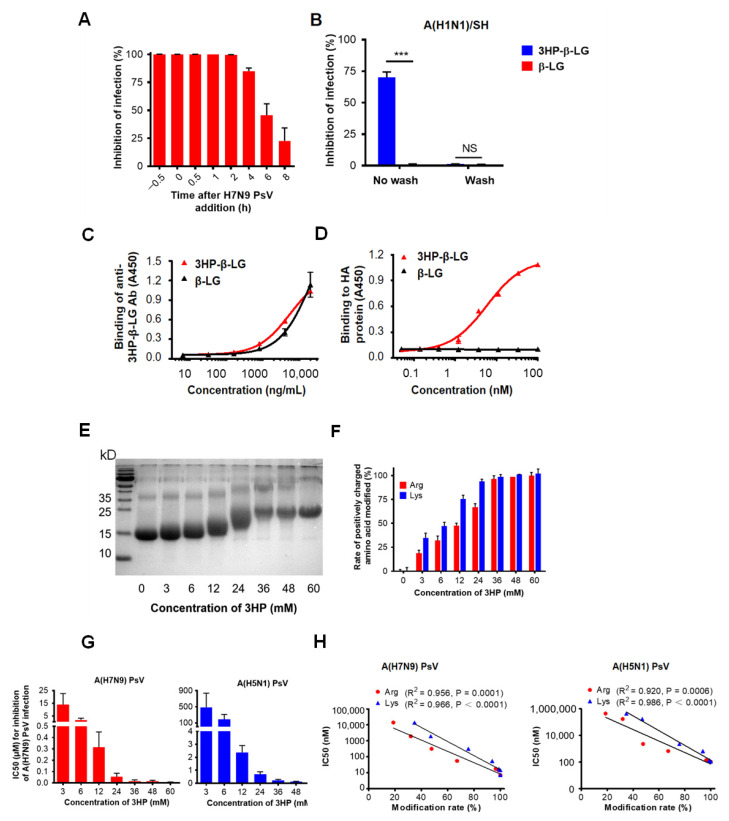
Mechanism of 3HP-β-LG inhibiting influenza virus infection. (**A**) Time-of-addition assay. Influenza A(H7N9) PsV was added into MDCK cells at 0 h and 3HP-β-LG was added at −0.5, 0, 0.5, 1, 2, 4, 6, and 8 h, respectively. Cell culture medium was substituted with fresh medium and tested 48 h post-infection. (**B**) Washout assay. After incubation with 3HP-β-LG, MDCK cells were not washed or were washed with DMEM before addition of influenza A(H1N1)/SH. Viral infectivity was measured as described in Materials and Methods. *** denotes *p* < 0.001; “NS” means no significance. (**C**) Binding of 3HP-β-LG and β-LG with anti-3HP-β-LG Ab as detected by ELISA. (**D**) Interaction of 3HP-β-LG and β-LG with influenza hemagglutinin (HA) protein detected by ELISA. The results are presented as mean ± SD. (**E**) SDS-PAGE analysis of β-LG modified with different concentration of 3HP. (**F**) Arginine modification rate (%) and lysine modification rate (%) of β-LG at different concentrations. (**G**) Inhibition of β-LG modified by different HP against influenza A(H7N9) and A(H5N1) PsVs. The “3 mM” means that β-LG was modified by 3HP at the concentration of 3 mM. Tests of different HP concentrations, including 3, 6, 12, 24, 36, 48, and 60 mM, respectively, were conducted and the inhibition of these concentrations against influenza A(H7N9) and A(H5N1) PsVs in MDCK cells was detected. Each sample was tested in triplicate and the data are displayed as mean ± SD. (**H**) The correlation between the modification rate of 3HP-β-LG and its antiviral activity (IC50) against influenza A(H7N9) PsV and A(H5N1) PsV is expressed by a linear equation. R^2^ and *p* values were calculated and are presented in the figure. Each sample was tested in triplicate and the data are shown as mean ± SD.

In order to investigate the mechanism of action of 3HP-β-LG, we performed a time-of-addition assay by adding the 3HP-β-LG to the cell culture at different time points before and after the addition of influenza A(H7N9) PsV, followed by detection of its inhibitory activity. As is shown in Figure 4A, the 3HP-β-LG-mediated inhibitory activity started to decrease when the 3HP-β-LG was added at 4 h post-infection, suggesting that 3HP-β-LG acts at the early stage of virus entry and infection.

In order to determine whether 3HP-β-LG acts on host cells, we performed a washout assay. For this procedure, 3HP-β-LG was preincubated with MDCK cells for 1 h and the cells were washed three times so as to remove the unbound 3HP-β-LG in the supernatant before the addition of influenza A(H1N1)/SH virus into the culture. In the control condition, the 3HP-β-LG-preincubated MDCK cells were not washed before the addition of the virus. As shown in Figure 4B, the MDCK cells were not protected from infection by the influenza virus if the cells were washed after incubation with 3HP-β-LG or β-LG; however, 3HP-β-LG, but not β-LG, exhibited inhibition against viral infection when the cells were not washed. A similar result was obtained when influenza A(H7N9) PsV was used in the washout assay, as was described above, indicating that 3HP-β-LG does not act on the receptor or other components of the surface of the host cells (Supplementary Figure S3).

HA is the main surface glycoprotein of the influenza virus that mediates both the binding of the virus with sialic acid on the host cell and the entry of virus into the host cell, thus serving as a target for the development of influenza virus entry inhibitors [31]. Therefore, we used ELISA to detect the interaction between 3HP-β-LG and influenza A(H7N9) virus HA using an anti-3HP-β-LG antibody that could effectively bind to 3HP-β-LG or β-LG (Figure 4C). The results show that 3HP-β-LG could bind to HA protein in a dose-dependent manner, while β-LG could not (Figure 4D). The observed interaction between 3HP-β-LG and the HA protein proved that 3HP-β-LG did act on virions, which resulted in blocking the binding between the HA protein and sialic acid receptor on the host cell’s membrane, thereby preventing viral invasion.

### 3.5. The 3HP-β-LG Bound through Its Negatively Charged Amino Acid Residues to Viral Positively Charged HA Protein

Our previous studies have shown that 3HP-β-LG acts on the virus, rather than the host cell, at the early stage of the virus’ life cycle, i.e., viral entry into the host cell, blocking the binding between the HA protein and sialic acid receptor on a host cell membrane. We have also shown that the inhibitory potency of 3HP-β-LG is correlated with the modification rate of the positively charged residues, Arg and Lys, confirming that 3HP-β-LG (with a reduced number of positively charged amino acid residues by modification with 3HP and, hence, relatively increased net negative charges) will interact with the positively charged residues on the HA protein of an influenza virus, resulting in the inhibition of viral entry and infection. Our studies have proved that this effect is dose-dependent and thus a determinant of the potency of the antiviral effect [4,19]. As proof-of-concept, we herein used a similar chemical modification assay to determine the correlation between the inhibitory activity of 3HP-β-LG against influenza virus infection and the percentage of positively charged amino acid residues that were modified. The Arg and Lys modification rates were determined based on the results from TNBS and HPG assays, respectively, as was previously described [24,25,26]. The bands of β-LG that were modified with different concentrations of 3-hydroxyphthalic anhydride (3HP) were located at different positions in the SDS-PAGE gel (Figure 4E). When a higher concentration of anhydride was used, a higher modification rate of the positively charged residues was achieved (Figure 4F) with correspondingly higher antiviral activity against infection of influenza A(H7N9) and A(H5N1) PsVs (Figure 4G). Therefore, the IC50 of 3HP-β-LG against influenza A(H7N9) and A(H5N1) PsV infection is reversely correlated with the modification rate of Arg and Lys (Figure 4H), confirming that the 3HP-modified β-LG (3HP-β-LG) with more positively charged amino acid residues that have been modified, or neutralized, by 3HP has a higher net negative charge on its surface and thus may interact with the positively charged residues on the viral surface protein (i.e., HA) of an influenza virus, thus possessing greater antiviral activity.

### 3.6. The 3HP-β-LG Exhibited Better Prophylactic Effect Than Therapeutic Effect against Influenza Virus Infection in C57BL/6 Mice

**Figure 5:**
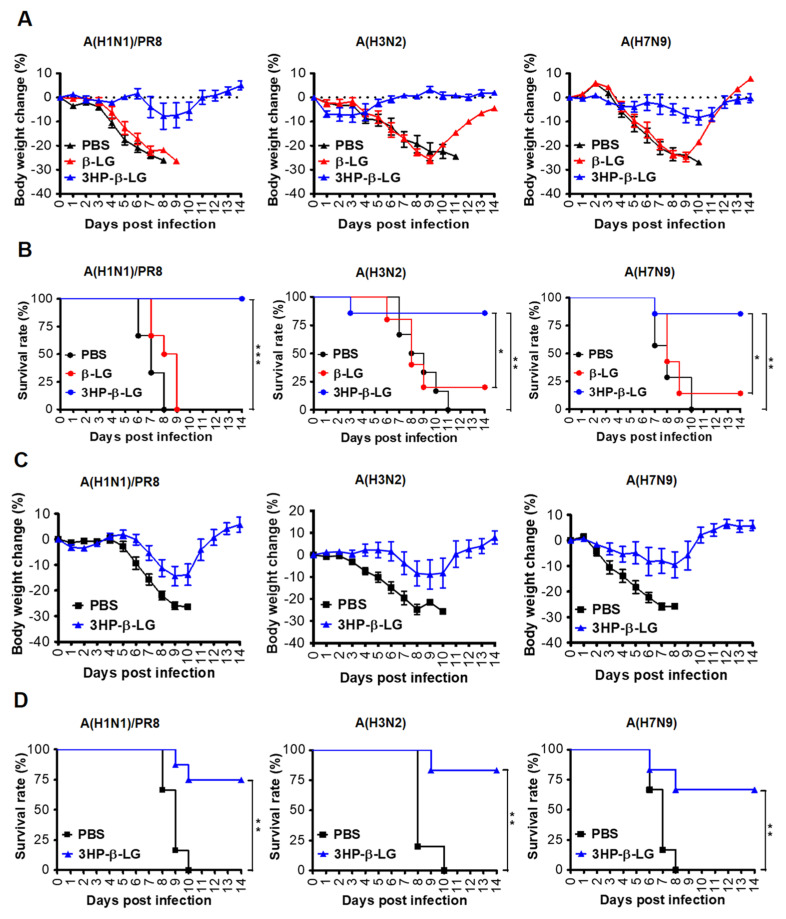
Protection of 3HP-β-LG against influenza virus infection in mice. (**A**,**B**) Prophylactic protection of 3HP-β-LG against influenza virus infection in mice treated with 3HP-β-LG 0.5 h before challenge with A(H1N1)/PR8, A(H3N2), or A(H7N9). (**A**) Bodyweight change (%) and (**B**) survival rate (%) of mice were recorded daily post-infection. *, **, and *** denote *p* < 0.05, 0.01, and 0.001, respectively. (**C**,**D**) Therapeutic protection of 3HP-β-LG against influenza virus infection in mice 4 h after challenge with A(H1N1)/PR8, A(H3N2), and A(H7N9). (**C**) Bodyweight change (%) and (**D**) survival rate of mice were recorded daily post-infection. Data are presented as mean ± SD.

In order to investigate the in vivo prophylactic effect of 3HP-β-LG, female *C57BL/6 mice* were administered with 3HP-β-LG (40 mg/kg), β-LG (40 mg/kg), or PBS via the nasal route. After 0.5 h, these treated mice were intranasally challenged with a lethal dose of an influenza virus, either influenza A(H1N1)/PR8, A(H3N2), or A(H7N9). The bodyweight losses (%) and survival rate (%) of the mice were recorded for 14 consecutive days. We found that mice that were administered with 3HP-β-LG had only slight bodyweight loss, while those with β-LG or PBS exhibited more obvious bodyweight loss (Figure 5A). The survival rates of the mice that were treated with 3HP-β-LG or β-LG and then challenged with the influenza A(H1N1)/PR8 virus were 100% and 0%, respectively. Those that were treated with 3HP-β-LG or β-LG and then challenged with the influenza A(H3N2) virus were 85.7% and 16.7%, respectively, while those that were administered with 3HP-β-LG or β-LG and then challenged with the influenza A(H7N9) virus were 85.7% and 14.3%, respectively (Figure 5B). The three virus-challenged mice that were administered with PBS had a 0% survival rate. These results suggest that, unlike β-LG and PBS, 3HP-β-LG is highly effective as a prophylactic agent in the protection of mice against influenza A(H1N1)/PR8, A(H3N2), and A(H7N9) infections.

In order to detect the therapeutic effect of 3HP-β-LG, mice were challenged with a lethal dose of influenza A(H1N1)/PR8, A(H3N2), or A(H7N9). After 4 h, 3HP-β-LG or PBS was intranasally administered as has been described above. The bodyweight losses and survival rate of the mice were monitored daily for 14 days. As is shown in Figure 5C, the 3HP-β-LG-treated mice that were challenged with influenza A(H1N1)/PR8, A(H3N2), and A(H7N9) viruses had less than 14% bodyweight loss and returned to normal about 9- and 12-days post-infection, respectively, while all of the mice that were treated with PBS showed more than 25% bodyweight loss about 8 to 10 days post-infection. The survival rates of the 3HP-β-LG-treated mice that were challenged with influenza A(H1N1)/PR8, A(H3N2), and A(H7N9) viruses were 75%, 83.3%, and 66.7%, respectively, while all of the PBS-treated and influenza virus-challenged mice had a 0% survival rate, suggesting that 3HP-β-LG also possesses a therapeutic effect against influenza A(H1N1)/PR8, A(H3N2), and A(H7N9) infections, although 3HP-β-LG’s therapeutic effect was less potent than its prophylactic effect (Figure 5D).

**Figure 6:**
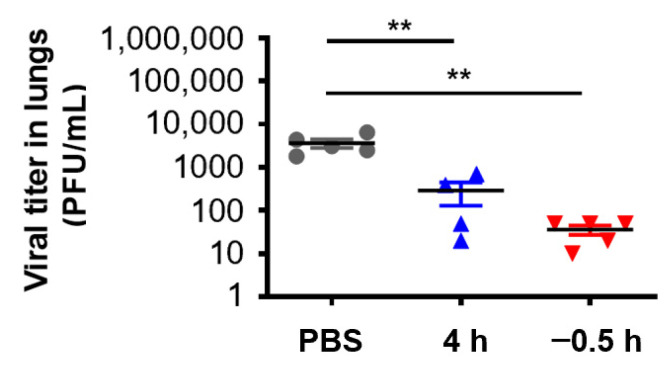
Virus titer in lungs of mice treated with 3HP-β-LG 0.5 h before or 4 h after viral challenge. ** denotes *p* < 0.01. Data are presented as mean ± SD.

In order to further verify the in vivo protective effect of 3HP-β-LG, we collected the lungs of the mice that were administered with 3HP-β-LG 0.5 h before or 4 h after they were challenged with influenza A(H1N1) or PR8, respectively, for the detection of viral titer. As is shown in Figure 6, the viral titers in the lung homogenates of the mice in the preventive group (administration 0.5 h before viral challenge) and treatment group (administration 4 h after viral challenge) were significantly lower than the viral titers in the PBS control group, indicating that 3HP-β-LG is effective in inhibiting influenza virus infection and replication in the mouse lung, thus protecting mice from death after infection.

## 4. Discussion

Bovine milk contains a number of whey proteins with biological significance in humans. For example, bovine lactoferrin (bLf) has been shown to inhibit infection of the influenza viruses H1N1 and H3N2 by interacting, through its C-lobe, with the highly conserved region containing the fusion peptide in viral HA, thus blocking viral fusion with host cells and preventing influenza virus-induced pathogenic effect [32,33]. In addition, bLf could significantly upregulate the expression levels of IFN-α, HLA-DR, and CD86 in pDCs, indicating that bLf is able to modulate the immune system by promoting pDC activation upon viral recognition [34]. Bovine β-LG, a globular protein that accounts for about 10% to 15% of total milk proteins, plays an important antioxidant role in milk [35,36], but no report shows its anti-influenza activity.

We previously demonstrated that 3HP-β-LG is a potent viral entry inhibitor against HIV, SARS-CoV-2, and human papilloma virus (HPV) [14,15,16,17,18,19]. In clinical trials, an anti-HPV biological dressing (JB01-BD) containing 3HP-β-LG was administered intravaginally (3 g per dose every other day for 3 months, except during the menstrual period) [14]. It was shown to be safe and effective in its ability to inhibit the entry and infection of HPV high-risk types in the cervical area and was thus approved for use in China starting in 2012 for vaginal use in treating patients with a cervical infection of high-risk type HPV [17]. So far, about 357,000 patients with high-risk type HPV infection have used JB01-BD and no severe adverse effect has been reported (unpublished data provided by Shanxi Jinbo BioPharmaceutical Co., Ltd. in Taiyuan, Shanxi Province, China). This suggests that 3HP-β-LG can be repurposed for local (e.g., intranasal) application in order to prevent infection by other viruses, such as influenza viruses.

In this study, we confirmed that 3HP-β-LG could also effectively inhibit IAV entry into the target cells and the in vitro infection of divergent influenza A and B viruses. Intranasal administration of 3HP-β-LG could protect mice from infection in vivo by the challenged influenza A(H1N1)/PR8, A(H3N2), and A(H7N9) viruses.

Mechanistic studies have shown that 3HP-β-LG acts on the virus, rather than the host cell, during the early stage of the virus’ life cycle, i.e., the viral entry into the host cell. Further study demonstrated that 3HP-β-LG could bind to HA protein in a dose-dependent manner, thus blocking the binding of the virions to the cellular receptor and their subsequent entry into the host cell. We have also shown that the inhibitory potency of 3HP-β-LG is correlated with the modification rate of the positively charged residues Arg and Lys, confirming that 3HP-β-LG (with a reduced number of positively charged amino acid residues by modification of 3HP and, hence, relatively increased net negative charges) acts on the positively charged residues of the HA of the influenza virus, resulting in the inhibition of viral entry and infection. This is consistent with the known mechanism of action of 3HP-β-LG for inhibiting the infection of HIV, HPV, and SARS-CoV-2 [14,15,16,17,18,19]. Subsequently, the safety of 3HP-β-LG was confirmed by in vitro cytotoxicity assay and in vivo animal experiment. Notably, very low-levels of IgG antibodies against 3HP-β-LG or β-LG were detected in the sera of the mice that were intranasally administrated with 3HP-β-LG or β-LG (Supplementary Figure S2B), suggesting that, in the absence of an adjuvant, local application of 3HP-β-LG may not elicit high-level anti-3HP-β-LG antibodies. However, it is unknown whether the repeated intranasal application of 3HP-β-LG in humans can induce IgA antibodies against 3HP-β-LG and whether the anti-3HP-β-LG IgA antibodies, if elicited, can attenuate 3HP-β-LG-mediated antiviral activity. Therefore, it is necessary to investigate this issue in future preclinical and clinical studies of 3HP-β-LG.

**Figure 7:**
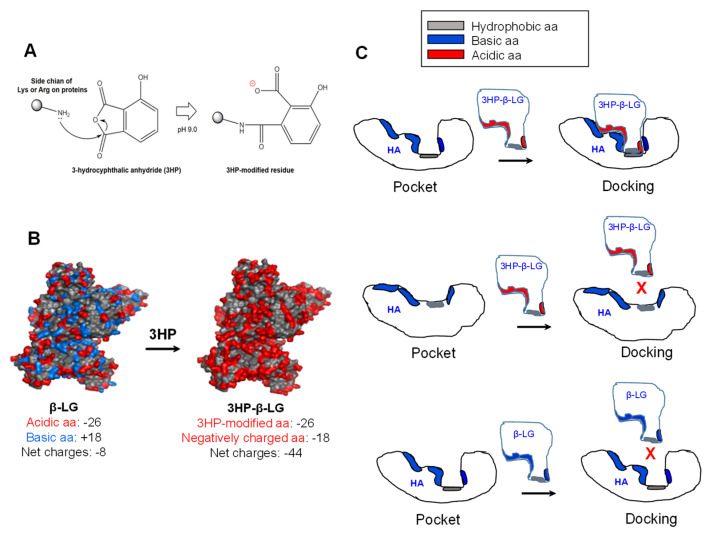
Modification of β-LG with 3HP and the binding model of 3HP-β-LG to the pocket region in viral HA. (**A**) Modification of a positively charged basic amino acid with 3HP results in generation of a negatively charged amino acid. (**B**) Modification of the positively charged basic amino acids in β-LG results in generation of 3HP-β-LG with much increased negatively charged amino acids. (**C**) 3HP-β-LG with enriched negatively charged residues can bind to the pocket in HA with matched conformation (upper panel), but cannot bind to the pocket in HA with non-matched conformation (middle panel). β-LG with enriched positively charged residues can bind to the pocket in HA with matched conformation (lower panel).

Why does 3HP-β-LG, but not β-LG, bind strongly to viral HA for inhibiting influenza virus infection? The modification of β-LG with 3HP through nucleophilic substitution chemical reactions is able to make the positively charged basic amino acids in β-LG become the negatively charged acidic amino acids that are found in 3HP-β-LG, resulting in a significant increase in the net negative charges that can enhance the binding affinity of 3HP-β-LG to viral HA (Figure 7A). For example, β-LG contains 26 negatively charged acidic amino acids (Asp and Glu) and 18 positively charged basic amino acids (Lys and Arg) and its net charge is −8. After the modification of β-LG with 3HP, the 18 positively charged basic amino acids become negatively charged amino acids, thus the net charge of 3HP-β-LG is −44, about 4.5-fold higher than that of β-LG (Figure 7B). Besides the interaction between the region in 3HP-β-LG with enriched negatively charged amino acid residues and the region in the viral HA with enriched negatively charged amino acid residues, the match of the conformations of the regions in 3HP-β-LG and HA that are used for docking is also important for their interaction. Otherwise, 3HP-β-LG and HA may not interact with each other even though 3HP-β-LG and HA have regions with enriched negatively and positively charged amino acid residues, respectively (Figure 7C). Therefore, it is essential to identify the region in HA that can interact with 3HP-β-LG, for better understanding the mechanism of action of 3HP-β-LG.

Anti-influenza virus antibodies may also be intranasally applied for the prevention of influenza virus infection. Compared with these antibodies, 3HP-β-LG has several advantages: (1) it can be used immediately at the beginning of the outbreak since it is expected to be effective against most influenza virus strains, while antibodies have to be tested for their efficacy against the influenza virus that causes the outbreak, (2) its production cost is expected to be much lower than that of antibodies, and (3) it can be stored and transported at room temperature, whereas antibodies must be stored and transported at low temperature. The disadvantages of 3HP-β-LG include: (1) people who are allergic to bovine proteins cannot use it and (2) it can only be used locally, e.g., intranasally or intravaginally, but not systemically in humans.

Influenza viruses have posed significant threats to global public health and emerging zoonotic influenza viruses may cause pandemics in the future. Because of the lack of broad-spectrum anti-influenza virus vaccines, 3HP-β-LG becomes a prime candidate as an intranasal prophylactic agent for the urgent prevention of infection by the influenza virus in order to stop its spread which may cause a global pandemic. Therefore, further development of 3HP-β-LG-based prophylactics is urgently needed.

## Data Availability

Not applicable.

## Supplementary Materials

The following supporting information can be downloaded at: https://www.mdpi.com/article/10.3390/v14092055/s1. Figure S1: Inhibition of 3HP-β-LG against influenza A(H1N1)/PR8 as detected by plaque reduction assay; Figure S2: (A) Bodyweight change (%) of mice intranasally administered with 3HP-β-LG or β-LG at 80 mg/kg, or PBS (as control), on day 0, 1, 3, and 5, respectively; Figure S3: Washout assay.
