# Supplementary material for: Chemically Modified Bovine β-Lactoglobulin as a Broad-Spectrum Influenza Virus Entry Inhibitor with the Potential to Combat Influenza Outbreaks
Source: Viruses. 2022 Sep 16;14(9):2055. doi: 10.3390/v14092055 (PMC9506557; doi:10.3390/v14092055)
Supplement: Supplementary file 1 [file viruses-14-02055-s001.zip › viruses-1867692-supplementary.pdf]

Supplemental Materials

Chemically Modified Bovine β-Lactoglobulin as a Broad-Spectrum Influenza Virus Entry Inhibitor with the Potential to Combat Influenza Outbreaks

Yuhong Fu [1], Peiyu Li [1,2], Wei Xu [1], Zezhong Liu [1], Cong Wang [1], Qian Wang [1], Jiayi Tang [1], Weihua Li [3], Lu Lu [1,*] and Shibo Jiang [1,2,3,*]

[1] Key Laboratory of Medical Molecular Virology of MOE/MOH, School of Basic Medical Sciences, Shanghai Public Health Clinical Center, Fudan University, 131 Dong An Rd., Xuhui District, Shanghai 200032, China
[2] Department of Infectious Diseases and Shenzhen Key Laboratory for Endogenous Infection, Shenzhen Nanshan People's Hospital and the Affiliated Shenzhen Sixth Hospital of Guangdong Medical University, Shenzhen 518052, China
[3] NHC Key Lab of Reproduction Regulation (Shanghai Institute for Biomedical and Pharmaceutical Technologies), Fudan University, 2140 Xie Tu Rd., Xuhui District, Shanghai 200032, China
* Correspondence: lul@fudan.edu.cn (L.L.); shibojiang@fudan.edu.cn (S.J.); Tel.: +86-21-5423-7671 (L.L.); +86-21-5423-7673 (S.J.)

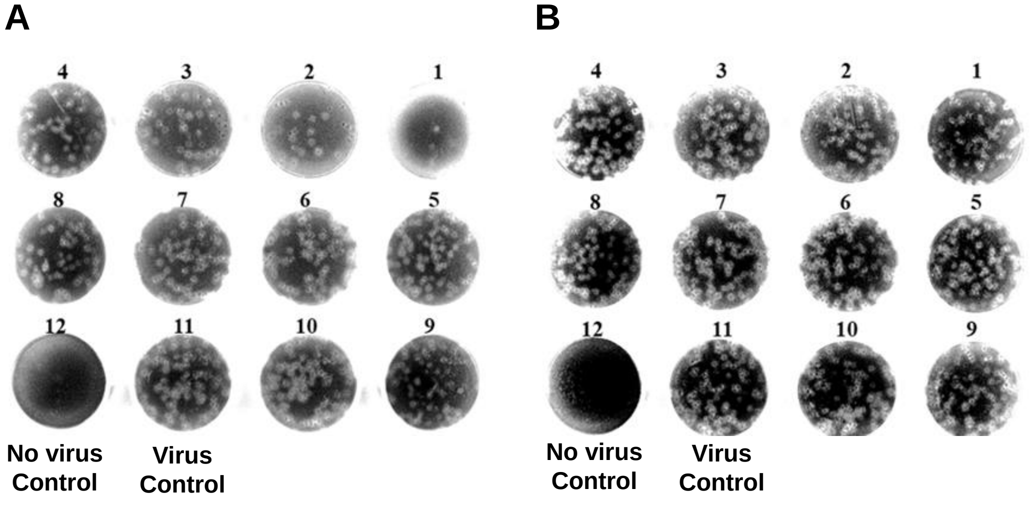

Group	Concentration (nM)										
No.	1	2	3	4	5	6	7	8	9	10	11	12
A (3HP-β-LG)	20000	2000	200	20	2	0.2	0.02	0.002	0.0002	0.00002	0	0
B (β-LG)	20000	2000	200	20	2	0.2	0.02	0.002	0.0002	0.00002	0	0
Virus	+	+	+	+	+	+	+	+	+	+	+	-

Figure S1: Inhibition of 3HP-β-LG against influenza A(H1N1)/PR8 as detected by plaque reduction assay

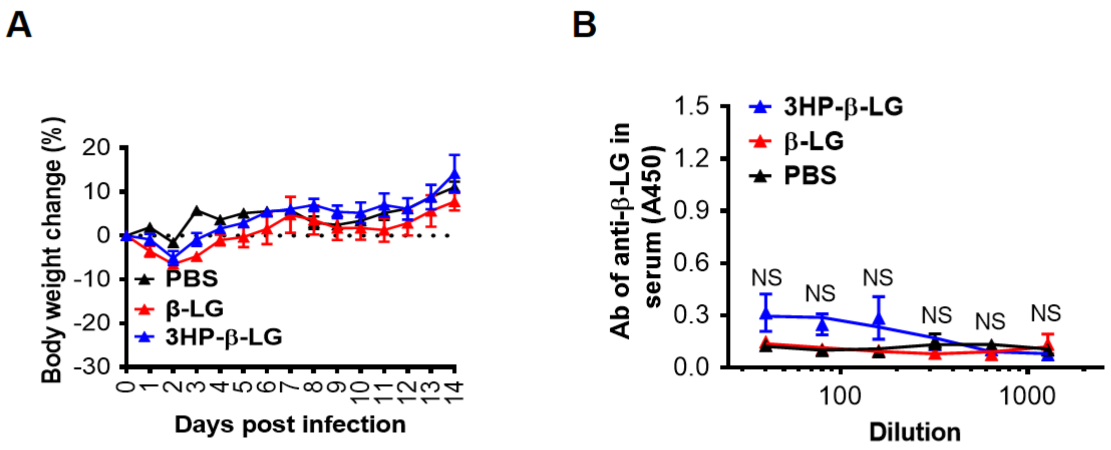

Figure S2: (A) Bodyweight change (%) of mice intranasally administered with 3HP-β-LG or β-LG at 80 mg/kg, or PBS (as control), on day 0, 1, 3, and 5, respectively. Bodyweight of mice was recorded d.p.i. (B) The titer of β-LG-specific antibodies in sera of mice intranasally administered with 3HP-β-LG or β-LG at 80 mg/kg, or PBS (as control), on day 0, 1, 3, and 5, respectively. Sera were collected and tested on day 14.

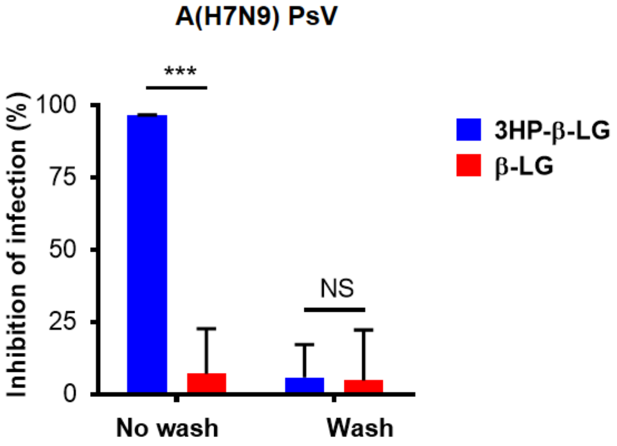

Figure S3: Washout assay. “Wash” or “No wash” indicate that MDCK cells treated with 3HP-β-LG or β-LG were washed or not washed before addition of influenza A (H7N9) PsV; and SDS-PAGE analysis of β-LG modified with different concentration of 3HP.
